# Controlling the surface‐mediated release of DNA using ‘mixed multilayers’

**DOI:** 10.1002/btm2.10023

**Published:** 2016-08-26

**Authors:** Visham Appadoo, Matthew C. D. Carter, David M. Lynn

**Affiliations:** ^1^ Dept. of Chemistry, 1101 University Avenue University of Wisconsin—Madison Madison WI 53706; ^2^ Dept. of Chemical and Biological Engineering, 1415 Engineering Drive University of Wisconsin—Madison Madison WI 53706

**Keywords:** gene delivery, layer‐by‐layer, multilayers, polymers, surfaces, thin films

## Abstract

We report the design of erodible ‘mixed multilayer’ coatings fabricated using plasmid DNA and combinations of both hydrolytically degradable and charge‐shifting cationic polymer building blocks. Films fabricated layer‐by‐layer using combinations of a model poly(β‐amino ester) (polymer **1**) and a model charge‐shifting polymer (polymer **2**) exhibited DNA release profiles that were substantially different than those assembled using DNA and either polymer **1** or polymer **2** alone. In addition, the order in which layers of these two cationic polymers were deposited during assembly had a profound impact on DNA release profiles when these materials were incubated in physiological buffer. Mixed multilayers ∼225 nm thick fabricated by depositing layers of polymer **1**/DNA onto films composed of polymer **2**/DNA released DNA into solution over ∼60 days, with multi‐phase release profiles intermediate to and exhibiting some general features of polymer **1**/DNA or polymer **2**/DNA films (e.g., a period of rapid release, followed by a more extended phase). In sharp contrast, ‘inverted’ mixed multilayers fabricated by depositing layers of polymer **2**/DNA onto films composed of polymer **1**/DNA exhibited release profiles that were almost completely linear over ∼60‐80 days. These and other results are consistent with substantial interdiffusion and commingling (or mixing) among the individual components of these compound materials. Our results reveal this mixing to lead to new, unanticipated, and useful release profiles and provide guidance for the design of polymer‐based coatings for the local, surface‐mediated delivery of DNA from the surfaces of topologically complex interventional devices, such as intravascular stents, with predictable long‐term release profiles.

## 1 Introduction

Thin films and coatings that promote the transfer of DNA to cells are important in a range of fundamental and applied contexts extending from the development of new research tools to the design of new platforms for the local delivery of DNA from implants and interventional devices.^1^, ^2^, ^3^, ^4^, ^5^, ^6^ These goals define fundamental challenges related to the integration of DNA with synthetic materials, the engineering of molecular assemblies with properties and behaviors governed by changes in weak interactions, and responsiveness to stimuli that are both complex and, in many cases, specific to the needs of a particular application. For example, while many applications may benefit from the rapid release of DNA from a surface (e.g., film‐coated microneedles for the delivery of DNA vaccines),^7^, ^8^, ^9^ others are likely to benefit from the gradual or sustained release of DNA over periods of days, weeks, or months (e.g., gene‐eluting intravascular stents).^1^, ^10^, ^11^ While many different materials can be used to immobilize DNA on surfaces,^1^, ^2^, ^3^, ^6^, ^12^, ^13^, ^14^, ^15^, ^16^, ^17^ the mechanisms and design rules that govern the assembly and disassembly of these materials—and the extents to which they influence the rates at which DNA is released and the forms in which it can be made available to cells—are poorly understood. The design of polymer‐based coatings that can be tuned to provide spatiotemporal control over the release of DNA remains an important goal as well as an obstacle to the development of new gene‐based therapies.

The work reported here takes steps toward addressing these challenges through (i) the design of ionically crosslinked polymer coatings (called ‘polyelectrolyte multilayers’) that erode in aqueous environments and promote the surface‐mediated release of DNA, and (ii) the development of new insights into the assembly of these materials that provide control over the rates at which these assemblies erode, disintegrate, and release DNA into surrounding media. Our approach is based on methods for the layer‐by‐layer assembly^13^, ^14^ of oppositely charged polymers on surfaces—a strategy that has been used in many past studies for the fabrication of thin, DNA‐containing coatings.^5^ Past studies have demonstrated that layer‐by‐layer assembly can be used to fabricate polyelectrolyte multilayers (e.g., ∼200 nm thick) using plasmid DNA and a variety of different natural and synthetic cationic polymers.^5^ When the cationic components of these assemblies contain structural features that can degrade or trigger changes in ionic interactions that destabilize the films, this approach provides a platform for the design of thin polymer films that erode and release their incorporated DNA ‘layers’. Many different cationic polymers have been developed for this purpose, with the extent to which DNA can be released rapidly, gradually, or selectively (e.g., in the presence of specific chemical triggers) dependent upon the types of chemical functionality incorporated into the polymer backbones or side chains.^5^, ^17^

When combined with other practical advantages of layer‐by‐layer assembly,^18^, ^19^ including control over film thickness, DNA loading and composition,^5^, ^20^ and the relative ease with which these methods can be used to fabricate conformal films on topologically complex objects (including the surfaces of common medical interventional devices),^21^, ^22^, ^23^ this ‘multilayered’ approach has the potential to be broadly useful for the development of new strategies for the localized delivery of DNA *in vitro* and *in vivo*.^24^, ^25^ However, while layer‐by‐layer assembly has been used widely in the basic research community to design coatings for the delivery or contact transfer of DNA,^5^, ^25^ there are currently no applications of these tools used in clinical practice. Potentially transformative applications continue to emerge,^7^, ^26^ but successful translation to clinical practice will require sustained efforts to understand the physicochemical properties and behaviors of these materials and the development of innovative new approaches to guide their design for specific applications.

**Figure 1:**
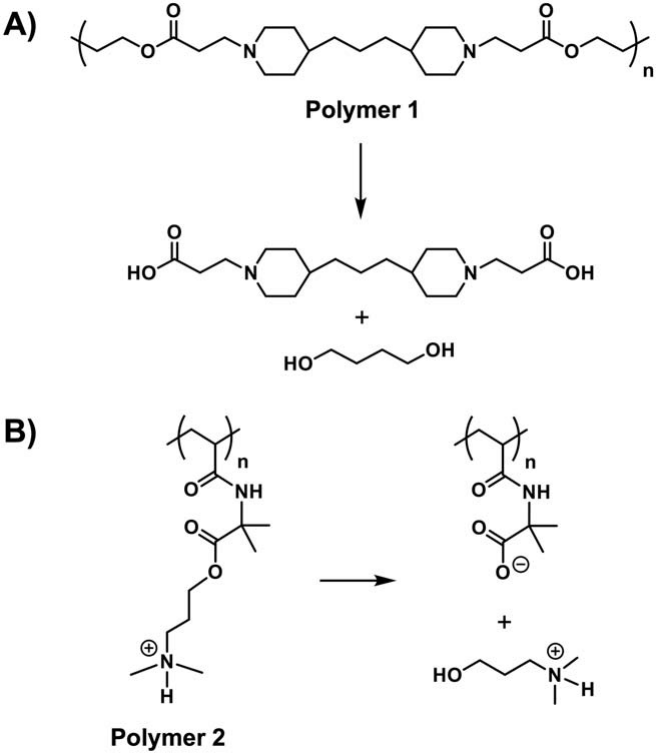
General scheme showing the structures of (A) polymer **1**, a hydrolytically degradable poly(*β*‐aminoester), and (B) polymer **2**, a charge‐shifting cationic polymer. The products resulting from backbone or side chain hydrolysis are also shown

The work reported here exploits two advances that have been used in past studies to promote and provide control over the disruption of polyelectrolyte multilayers. Our group has reported extensively on the utility of a class of hydrolytically degradable polyamines called poly(β‐amino ester)s (Figure 1A) as building blocks for the assembly of erodible plasmid DNA‐containing coatings.^22^, ^23^, ^27^ In this approach, film disassembly is driven, at least in part, by gradual backbone ester hydrolysis,^28^ and the release of DNA generally occurs over short periods (e.g., over a period of several days upon exposure to physiologically relevant media^23^, ^27^). As an alternative to the use of hydrolytically degradable polyamines, we^29^, ^30^, ^31^ and others^32^, ^33^, ^34^, ^35^ have also reported on the design of cationic ‘charge‐shifting’ polymers. These polymers are not backbone degradable, but instead contain hydrolyzable ester‐based side chains (Figure 1B) that lead to changes in polymer net charge that can, in turn, lead to the disruption of ionic assemblies (e.g., by becoming less positively charged over time). In contrast to approaches based on the incorporation of poly(β‐amino ester)s, this approach leads to thin polymer coatings that can erode and promote the surface‐mediated release of plasmid DNA for prolonged periods (e.g., over periods of up to several months).^29^, ^31^

In this study, we sought to explore the behaviors of ‘mixed’ multilayers fabricated using DNA and combinations of both poly(β‐amino ester)s and charge‐shifting cationic polymers. This work was broadly motivated by the hypothesis that films fabricated using combinations of these two different materials could open the door to new designs and film compositions that exhibit unique and useful release profiles that cannot be attained using coatings constructed from either type of polymer alone (e.g., coatings that permit the staged or sequential release of DNA, or release profiles with other useful features that can be tuned, accentuated, or eliminated by varying film composition and structure).

The results reported here demonstrate that changes in the nominal structures and compositions of coatings fabricated using combinations of poly(β‐amino ester)s and charge‐shifting cationic polymers lead to large changes in the behaviors (e.g., the release profiles) of these materials when exposed to physiologically relevant media. In particular, we find that the order in which layers of these two polymers are deposited (e.g., whether DNA and poly(β‐amino ester) layers are deposited before layers of DNA and charge‐shifting polymers, or *vice versa*) has profound effects on release behaviour, leading, in some cases, to films that can promote the extended release of one or more plasmid DNA constructs with linear release profiles that are difficult to obtain using other polyelectrolyte multilayer systems. Our results are consistent with processes of interdiffusion and mixing that occur among polymer layers during the assembly or erosion of these materials, and provide guidance useful for the design of conformal thin‐film coatings that can promote the release of DNA from surfaces at constant rates for up to 80 days. These methods expand the range of DNA release profiles that can be accessed using a limited pool of layer‐by‐layer building blocks and are, in principle, substrate independent and amenable to the immobilization and subsequent long‐term release of DNA from the surfaces of interventional devices such as intravascular stents.

## 2 Materials and methods

### 2.1 Materials

Diazabicycloundec‐7‐ene (DBU, 98%), concentrated hydrochloric acid (HCl, 37%, ACS reagent), azobisisobutyronitrile (AIBN, recrystallized from methanol), tetrahydrofuran (THF, HPLC grade), dichloromethane (DCM, ACS grade), hexanes (technical grade), 4,4′‐trimethylenedipiperidine (97%), sodium poly(4‐styrenesulfonate) (SPS, M_w_ = 70,000), and ethyl acetate (ACS grade, >99.5%) were purchased from Sigma‐Aldrich (Milwaukee, WI). 3‐Dimethylamino‐1‐propanol (99%) was purchased from Acros Organics (Morris Plains, NJ). 1,4‐Butanediol diacrylate was purchased from Alfa Aesar (Ward Hill, MA). Anhydrous THF was obtained from a Pure Process Technology solvent purification system (Nashua, NH). Linear poly(ethyleneimine) (LPEI, MW = 25000) was obtained from Polysciences, Inc. (Warrington, PA). Inhibitor Removal Resin was purchased from Alfa Aesar (Radnor, PA). 2‐Vinyl‐4,4‐dimethylazlactone (VDMA), a kind gift from Dr. Steven M. Heilmann (3M Corp., Minneapolis, MN) was fractionally distilled under vacuum (B.P. ∼22°C at ∼500 mTorr; clear mobile liquid at room temperature) and then stored with 500 ppm butylated hydroxytoluene (BHT) and 1000 ppm triethylamine at 0°C prior to use. Polymer **1** (M_n_ = 23.3 kDa, *Ð* = 2.39) was synthesized as previously described.^36^ Phosphate‐buffered saline (PBS, pH = 7.4, ionic strength = 154 mM) was prepared by dilution of commercially available concentrate (EM Science, Gibbstown, NJ). Plasmid DNA [pEGFP‐N1 (encoding enhanced green fluorescent protein; EGFP) and pDsRed2‐N1 (encoding red fluorescent protein; RFP; pRFP) (4.7 kb), >95% supercoiled] was obtained from Elim Biopharmaceuticals, Inc. (San Francisco, CA). Dulbecco's modified Eagle's medium (DMEM), fetal bovine serum (FBS; 10%), penicillin (100 units/mL), streptomycin (100 μg/mL), Opti‐MEM reduced serum medium, trypsin‐EDTA (0.25%), and Lipofectamine 2000 were purchased from Invitrogen (Carlsbad, CA). COS‐7 cells were obtained from the American Type Culture Collection (Manassas, VA). Expired stainless steel stents used for SEM studies (Abbott Vascular, Abbott Park, IL; Medtronic, Shoreview, MN) were obtained from the Cardiovascular Physiology Core Facility at the University of Wisconsin‐Madison. All stents had nominal diameters ranging from 2 to 5 mm and lengths ranging from 8 to 30 mm. Stent expansion was performed using a standard inflation device with deionized water as the expansion fluid. All buffers and polymer solutions were filtered through a 0.2 μm membrane syringe filter prior to use unless noted otherwise. Test‐grade n‐type silicon wafers were purchased from Silicon Inc. (Glenshaw, PA). Water with a resistivity of 18.2 MΩ was obtained from a Millipore filtration system. Materials were used as received unless otherwise noted.

### 2.2 General considerations

^1^H NMR spectroscopy was performed in CDCl_3_ or D_2_O using a Bruker Avance‐400 or Bruker Avance‐500 spectrometer and a pulse repetition delay of 10 s. All spectra were referenced relative to the residual proton peak of CHCl_3_ (*δ* 7.26 ppm) or D_2_O (*δ* 4.79 ppm). Size exclusion chromatography (SEC) analyses for polymer **1** were performed using a Waters L9 515 344M GPC equipped with two Styragel HT6E columns (300 mm x 7.8 mm), a Waters 515 HPLC pump, a Waters 7726i manual injector, and a Waters 2410 RI detector, using THF with 0.1 M NEt_3_ as the eluent at a flow rate of 1 mL/min at 40°C. SEC analyses for poly(2‐vinyl‐4,4‐dimethylazlactone) (PVDMA) were performed using a Viscotek GPC Max VE2001 equipped with two Polymer Laboratories PolyPore columns (250 mm × 4.6 mm) and a TDA‐302 detector array using THF as the eluent at a flow rate of 1 mL/min at 40°C. The SEC instruments were both calibrated using 10 narrow dispersity polystyrene standards with *M*_n_ = 0.580 − 377.4 kg/mol (Agilent Technologies, Santa Clara, CA). Attenuated total reflectance (ATR) IR measurements were obtained using a Bruker Tensor 27 FTIR spectrometer outfitted with a Pike Technologies Diamond ATR stage (Madison, WI). Data were analyzed using Opus Software version 6.5 (Bruker Optik GmbH). Spectra were collected at a resolution of 2 cm ^−^ 1 and are presented as an average of 16 scans. Data were smoothed by applying a nine‐point average and baseline‐corrected using a concave rubberband correction (10 iterations, 64 points). Optical thicknesses of films deposited on silicon substrates were determined using a Gaertner LSE ellipsometer (632.8 nm, incident angle = 70°) and data were processed using the Gaertner ellipsometer measurement software. Thicknesses were calculated using a pre‐determined refractive index for each location measured on the film, and were determined in at least five different locations for three replicate films. All films were dried under a stream of filtered compressed air prior to thickness measurements. Scanning electron micrographs were acquired using a LEO‐1550 VP field‐emission SEM operating with an accelerating voltage of 1‐3 kV. Samples were coated with a thin layer of gold prior to imaging. UV/Vis absorbance values for solutions used to characterize DNA release profiles were recorded on a Beckman Coulter DU520 UV/Vis spectrophotometer (Fullerton, CA). Fluorescence microscopy images used to evaluate EGFP or RFP expression in transfection experiments were acquired using an Olympus IX70 microscope equipped with a Lumen Dynamics XCite 120PC‐Q fluorescence source and a QImaging EXi Aqua camera. Images were analyzed and false‐colored using MetaMorph Advanced software, Version 7.7.8.0 (Molecular Devices, LLC).

### 2.3 Synthesis of poly(2‐vinyl‐4,4‐dimethylazlactone) (PVDMA)

2‐Vinyl‐4,4‐dimethylazlactone was passed twice through a short column of Inhibitor Removal Resin followed by a short column of silica gel to remove inhibitor and triethylamine base, respectively. VDMA (2.06 g, 14.8 mmol), AIBN (24.32 mg, 0.148 mmol), and ethyl acetate (6.0 mL, dried twice over MgSO_4_) were added to an oven‐dried 25 mL round‐bottomed flask tube and sparged with nitrogen for 15 minutes before being placed into an oil bath at 60°C. After 24 hours, the flask was cooled to room temperature and the mixture was diluted with ∼4 mL of DCM and precipitated into ∼150 mL of hexanes. The resulting white solid was collected by vacuum filtration, redissolved in DCM, re‐precipitated twice more in hexanes, and then dried under high vacuum overnight. M_n_ = 47.6 kDa; *Ð =* 3.64. ^1^H NMR (400.180 MHz, CDCl_3_, *δ* ppm): 2.71 (s, 1H), 2.16‐1.79 (m, 2H), 1.37 (s, 6H). ATR IR (cm^−1^): 1818 (C = O azlactone), 1671 (C = N azlactone), 1203 C‐O‐C (azlactone); peaks at ∼3200 cm ^−^ 1 (NH stretch) and ∼1540 cm ^−^ 1 (NH bend) absent, indicating no unintended azlactone hydrolysis by adventitious water.

### 2.4 Synthesis of charge‐shifting polymer 2

Charge‐shifting polymer **2** was synthesized based on a modified literature procedure,^29^, ^31^ as follows. Briefly, PVMDA (0.512 g, 3.68 mmol), 3‐dimethylamino‐1‐propanol (1.3 equiv. with respect to the azlactone groups of PVDMA, 557.4 µL, 4.78 mmol) and DBU (55.1 µL, 0.368 mmol, 0.1 equiv. with respect to the azlactone groups of PVDMA) were dissolved in 8.0 mL of anhydrous THF in a 15 mL glass vial. The vial was sealed with a Teflon cap and parafilm and stirred at 65°C overnight. The resulting light yellow solution was concentrated under reduced pressure to ∼5 mL total volume and precipitated into 100 mL of 1:1 (v/v) hexanes/acetone containing 307 µL of concentrated HCl_(aq)_ (1 equiv. with respect to VDMA). The resulting sticky white solid was isolated by centrifugation, dried in air, dissolved in ∼8 mL MeOH, and precipitated into 150 mL of 1:1 (v/v) hexanes/acetone containing 307 µL of concentrated HCl_(aq)_ (37%). This process was repeated a total of six times to remove the excess unreacted alcohol (as monitored by ^1^H NMR in D_2_O) to yield the product as a slightly sticky white solid after drying under high vacuum overnight. ^1^H NMR (500.022 MHz, 1.0 mL D_2_O and 10 μL DCl, *δ* ppm): 4.35 (s, 2H, ‐CH_2_‐CH_2_‐CH_2_‐COC(O)‐), 3.28 (s, 2H, ‐CH_2_‐CH_2_‐CH_2_‐COC(O)‐), 2.93 (s, 6H, ‐N(CH_3_)_2_)‐CH_2_‐), 2.16 (s, 2H, CH_2_‐CH_2_‐CH_2_‐ COC(O)‐), 1.52 [m, 3H, ‐COC(O)‐C(CH_3_))_2_, and 6H, (‐CH_2_CH‐)]. ATR‐IR (cm ^−^ 1): 3350 (NH stretch), 1732 (C = O ester), 1658 (C = O amide), 1151 (C‐O‐C ether).

### 2.5 Preparation of polyelectrolyte solutions

Solutions of LPEI and SPS used for the fabrication of LPEI/SPS base layers^27^ were prepared at a concentration of 20 mM (with respect to the molecular weight of the polymer repeat unit) in aqueous solutions containing 10 mM NaCl. LPEI solutions also contained 5 mM HCl to aid polymer solubility. Solutions of polymers **1** and **2** were prepared at a concentration of 5 mM (w.r.t the molecular weight of the polymer repeat unit) in 100 mM sodium acetate buffer (pH = 5.0). All polymer solutions were filtered through a 0.2 μm nylon syringe filter prior to use. Solutions of plasmid DNA were prepared at concentrations ranging from 0.7‐1.0 mg/mL in 100 mM sodium acetate buffer (pH = 5.0) and were not filtered prior to use.

### 2.6 Fabrication of polyelectrolyte multilayers

**Figure 2:**
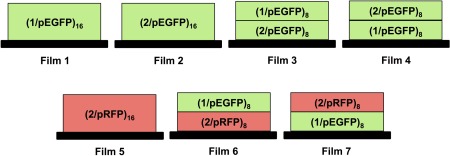
Schematic representations of the polyelectrolyte multilayer film architectures investigated in this study. Polymer **1** and polymer **2** are denoted here as ‘**1**’ and ‘**2**’, respectively. Plasmid DNA encoding green fluorescent protein and red fluorescent protein are denoted ‘pEGFP’ and ‘pRFP’, respectively, and sets of layers fabricated using these two plasmids are colored green or red coded for additional emphasis. Subscripts indicate the number of bilayers of cationic polymer and DNA that were deposited

Prior to use, silicon substrates (0.5 × 3.5 cm) were rinsed with acetone, ethanol, and deionized water and then dried under a stream of filtered compressed air. Stainless steel stents were used as received. Films designed to contain polymer **1** in the bottommost layers (e.g., Films 1, 4, and 7; see Figure 2) were first pre‐coated with a multilayer film composed of 10 bilayers of LPEI and SPS (terminated with SPS), as previously described using an automated dipping robot (Riegler & Kirstein GmbH, Potsdam, Germany).^23^ Films designed to contain polymer **2** in the bottom‐most layers (see Figure 2) were deposited directly onto the substrates without any precursor layers. Films were deposited on bare or pre‐coated substrates manually using the following general protocol: 1) substrates were submerged in a solution of polymer **1** or polymer **2** for 5 min, 2) substrates were removed and immersed in either 100 mM sodium acetate buffer at pH = 5.0 (after the deposition of polymer **1**) or water (after the deposition of polymer **2**) for 1 min followed by a second similar rinse for 1 min, 3) substrates were submerged in a solution of DNA for 5 min, and 4) substrates were rinsed in the manner described above. This cycle was repeated until the desired number of polymer/DNA layers (or ‘bilayers’) was reached. We describe films fabricated in this manner using the notation (X/DNA)_n_, where “X” refers to the polymer used and “n” denotes the number of polyamine/DNA bilayers deposited. The nominal structures of films and ‘mixed multilayer’ architectures investigated in this study are depicted in Figure 2. For experiments aimed at characterizing film growth profiles by ellipsometry, films were dried after every two cycles using filtered compressed air prior to characterization. Films to be used in erosion experiments were either used immediately or dried under a stream of filtered compressed air and stored in a vacuum desiccator until use. All films were fabricated at ambient room temperature. Films fabricated on stents and used in erosion studies were coated on both the inner and outer portions of the stent in the unexpanded state. Following film fabrication, stents were expanded by mounting them onto catheter balloon deployment systems. The catheter balloons were inflated to expand the stents and then deflated and removed. Stents used for SEM imaging studies were mounted and crimped onto catheter balloons prior to coating with multilayered films.

### 2.7 Characterization of film erosion and DNA release profiles

Experiments designed to characterize film erosion and DNA release profiles were performed in the following general manner.^27^ Film‐coated substrates were placed into a plastic cuvette and 1.0 mL of PBS solution was added to cover the film‐coated portion of the substrate. The samples were incubated at 37°C and transferred to fresh PBS solutions at predetermined intervals. UV/Vis absorbance readings were made directly on the incubation solutions and measurements were used to determine the amount of DNA released (λ = 260 nm).

### 2.8 Cell transfection assays

COS‐7 cells were grown in 96‐well plates for 24 hours at an initial seeding density of 12,000 cells/well in 200 μL of growth medium (90% DMEM, 10% FBS, penicillin 100 units/mL, streptomycin 100 μg/μL). After 24 hours, 10 μL of a Lipofectamine 2000/DNA plasmid mixture was added directly to each well according to the general protocol provided by the manufacturer (experiments were performed in triplicate). The Lipofectamine 2000/DNA plasmid mixtures used contained 25 μL of DNA solution collected at a given time point during a release experiment and 25 μL of Lipofectamine 2000 reagent (24 μL stock diluted into 976 μL of water). Fluorescence images used to qualitatively characterize levels of gene expression were acquired after 48 hours.

## 3 Results

### 3.1 Fabrication of mixed multilayers: deposition of degradable polymer 1/DNA layers on charge‐shifting polymer 2/DNA films

**Figure 3:**
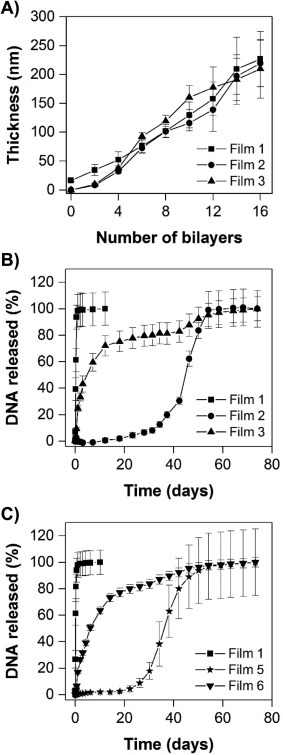
(A) Plot of film thickness versus the number of polymer/DNA layers deposited on silicon substrates as determined by ellipsometry during the growth of Films 1, 2, and 3 (see Figure 2 for additional details of the compositions of these films). (B) Plot showing the release of DNA from substrates coated with Films 1, 2, and 3. (C) Plot showing the release of DNA from substrates coated with Films 1, 5, and 6. (B‐C) Films were incubated in PBS buffer (pH 7.4, 37°C) and the amount of DNA released into solution was measured by UV/Vis absorbance. In each case, data are presented as the average and standard deviation of measurements obtained on three identically prepared films

We performed an initial series of experiments to characterize the fabrication and film growth profiles of ‘mixed multilayers’ assembled using a combination of poly(β‐amino ester) **1** and charge‐shifting polymer **2** as cationic film components together with a plasmid DNA construct encoding EGFP as an anionic building block. For these experiments, we fabricated films having the general structure represented by Film 3 shown in Figure 2 by depositing eight bilayers of polymer **1** and DNA onto a multilayer film composed of eight bilayers of polymer **2** and DNA (e.g., a film having the nominal structure (polymer **2**/pEGFP)_8_(polymer **1**/pEGFP)_8_; see Materials and Methods for additional details of film fabrication procedures). Figure 3A (closed triangles) shows a plot of film thickness versus the number of polymer and DNA layers deposited on a planar silicon substrate during fabrication. Inspection of these results reveals film growth to occur in a linear manner that is characteristic of the iterative, layer‐by‐layer growth of control films fabricated exclusively from pEGFP and either polymer **1** [i.e., (polymer **1**/pEGFP)_16_, Film 1 in Figure 2; closed squares in Figure 3)]^27^ or polymer **2** [i.e., (polymer **2**/pEGFP)_16_, Film 2 in Figure 2; closed circles in Figure 3)]^29^ to an optical film thickness of ∼225 nm. These results demonstrate that ‘hydrolytically degradable’ bilayers of polymer **1**/pEGFP can be deposited directly on the surfaces of ‘charge shifting’ films fabricated from polymer **2** and DNA without any significant changes in film growth profiles (e.g., without a change in slope or a transition from linear to a phase reflecting so‐called exponential growth,^37^, ^38^, ^39^, ^40^ etc.).

### 3.2 Release profiles of mixed (polymer 2/DNA)–(polymer 1/DNA) multilayers in aqueous buffer

We characterized the release profiles of mixed (polymer **2**/pEGFP)_8_ (polymer **1**/pEGFP)_8_ multilayers in physiologically relevant media by incubating them in PBS buffer at 37°C and characterizing the release of pEGFP into solution over time. Figure 3B (closed triangles) shows the release of DNA from these Film 3‐coated silicon substrates in comparison to the release of DNA from control substrates coated with either (polymer **1**/pEGFP)_16_ films (Film 1; closed squares) or (polymer **2**/pEGFP)_16_ films (Film 2; closed circles). Inspection of these results reveals substrates coated with Film 1 to release DNA into solution rapidly (e.g., over ∼1‐2 days), consistent with the behaviors of these hydrolytically degradable coatings reported in past studies.^23^, ^27^ In contrast, substrates coated with Film 2 exhibited prolonged release profiles, with an extended ‘lag’ phase of ∼2‐3 weeks, followed by the onset of release extended up to ∼60 days under these conditions; this behavior is also consistent with those of past studies of films fabricated using DNA and charge‐shifting polymer **2**.^29^, ^31^ Substrates coated with Film 3 [so‐called ‘mixed’ (polymer **2**/pEGFP)_8_(polymer **1**/pEGFP)_8_ multilayers] exhibited release profiles that were intermediate to those two extremes. As shown in Figure 3, Film 3 (closed triangles) also released DNA over an extended period of ∼60 days. These films did not exhibit a delayed ‘lag’ phase typical of the Film 2 architecture or release a substantial percentage of their DNA over the first 1‐2 days of immersion in physiological media.

### 3.3 Fabrication and characterization of mixed multilayers on intravascular stents

**Figure 4:**
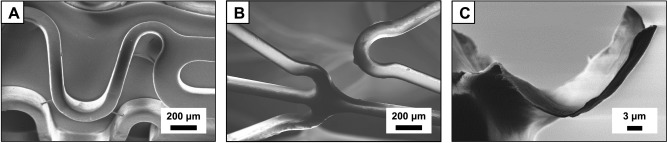
Scanning electron microscopy images showing intravascular stents coated with Film 3 (see Figure 2 for additional details of the compositions of these films). (A) Film‐coated stent crimped on a catheter balloon prior to expansion. (B) Film‐coated stent after expansion and removal of the catheter balloon. (C) High‐magnification image showing a region the film that was partially delaminated from the stent surface; images such as those in (C) were used to characterize film thickness, see Figure S1

Panels A‐B of Figure 4 show SEM images of bare metal stainless steel intravascular stents coated with mixed Film 3 multilayers after hand‐crimping onto a deflated balloon assembly (A) and after inflation of the balloon to re‐expand the stent (B). These images reveal the surfaces of the stents to be coated with a uniform and conformal polymer/DNA film that did not crack, peel, or delaminate substantially from the surface of the stent over large areas when subjected to mechanical forces associated with crimping or balloon expansion. Figure 4C shows a selected portion of a film from a small region of a coating that did partially delaminate from the stent surface; cross‐sectional analysis of this and other related images (see Figure S1) reveals these mixed Film 3 stent coatings to be ∼240 (±70) nm thick, a value that is in general agreement with optical thicknesses measured using ellipsometry for films fabricated on silicon substrates (Figure 3A). These Film 3‐coated stents also released pEGFP gradually over extended periods of ∼60 days when incubated in PBS buffer at 37°C (Figure S2), with an overall release profile that was similar to that shown in Figure 3B for Film 3‐coated silicon substrates (and intermediate to those exhibited by control stents coated with Film 1 or Film 2).

### 3.4 Characterization of mixed multilayers fabricated using two plasmid constructs

To provide additional insight into the release profiles of the Film 3 structures described above, we fabricated films having the structure shown in Film 6 of Figure 2 [i.e., (polymer **2**/pRFP)_8_(polymer **1**/pEGFP)_8_] using polymer **1**, polymer **2**, and two different plasmid DNA constructs encoding either EGFP or RFP. These films were fabricated in a manner identical to that used to fabricate Film 3, with the exception that pRFP was used to fabricate the bottommost portion of the films (i.e., the (polymer **2**/pRFP) bilayers). Silicon substrates coated with Film 6 exhibited DNA release profiles nearly identical to those of substrates coated with Film 3 [(see Figure 3C, closed triangles; the release profile of substrates coated with control films fabricated using polymer **2** and the pRFP plasmid (Film 5; Figure 3, closed stars) is shown for comparison, and is similar to that of substrates coated with polymer **2** and the pEGFP plasmid; Figure 3B)]. These results demonstrate that this second pRFP plasmid, which is similar in size to the pEGFP plasmid (4.7 kb), does not have a significant impact on the erosion and DNA release profiles of these mixed multilayer films (coatings with the Film 6 structure were ∼250 nm thick, as determined by ellipsometry, and were thus also similar in thickness to the Film 3 coatings).

**Figure 5:**
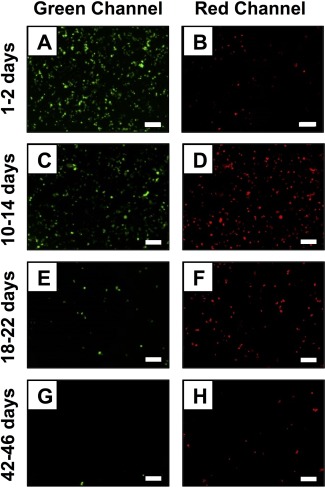
Representative fluorescence microscopy images showing COS‐7 cells expressing GFP (green channel) and RFP (red channel) after treatment with samples of DNA collected during the erosion of substrates coated with Film 6 (see Figure 2 for additional details of the compositions of these films; see main text and Materials and Methods for additional details for these transfection experiments). The relative levels of GFP and RFP expression observed correspond qualitatively to the amount of each plasmid released from the film during the following time periods: (A‐B) 1‐2 days, (C‐D) 10‐14 days, (E‐F) 18‐22 days, and (G‐H) 42‐46 days. Scale bar = 250 μm. A complete set of images for all time points measured is provided in Figure S1

To determine whether the structures of these mixed multilayers could be used to provide differential control over the release of the pEGFP and pRFP plasmid constructs (e.g., whether both plasmids were released simultaneously or in a manner that was sequential or ‘staged’), we collected samples of DNA released over defined intervals during the course of the film erosion experiments described above and used them to transfect mammalian COS‐7 cells (using a commercial lipid as a delivery agent; see Materials and Methods and past studies^27^, ^29^, ^30^, ^31^, ^41^ for additional details of this experimental design). These studies were not aimed at quantifying relative levels of transgene expression, but rather to provide a qualitative, expression‐based indicator of the presence or absence of either plasmid in the degradation milieu at any time interval.^30^, ^31^, ^41^ We note here that films fabricated using fluorescently labeled plasmids were not used for these studies on the basis of past studies demonstrating that that approach is not reliable for the characterization of DNA in experiments requiring long incubation times (e.g., over 60 days).^31^ The results of studies using Film 6 coatings are summarized in Figure 5 for samples of DNA collected over four time periods early (over 1‐2 days of incubation; A‐B), mid‐way (over 10‐14 days or 18‐22 days; C‐D and E‐F, respectively) or late (over 42‐46 days; G‐H) in the release experiment (a complete set of results for samples collected over other intermittent time periods during this experiment can be found in Figure S3). Inspection of these results and those shown in Figure S3 reveals that EGFP is expressed almost exclusively in samples collected during the first ∼48 hours, and that both EGFP and RFP are expressed simultaneously at subsequent time points. We return to these observations again in the discussion below.

### 3.5 Characterization of ‘inverted’ mixed multilayers: long‐term linear release of DNA

**Figure 6:**
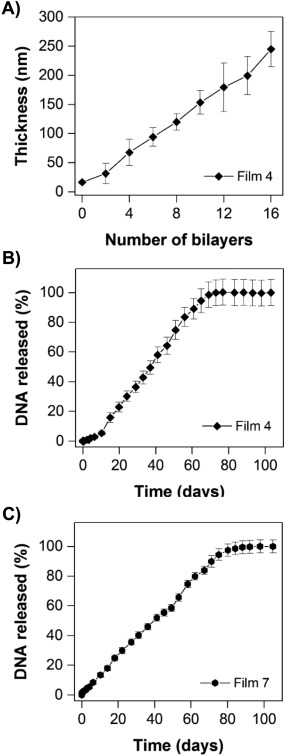
(A) Plot of film thickness versus the number of polymer/DNA layers deposited on silicon substrates as determined by ellipsometry during the growth of Film 4. (B) Plot showing the release of DNA from substrates coated with Film 4. (C) Plot showing the release of DNA from substrates coated with Film 7. (B‐C) Films were incubated in PBS buffer (pH 7.4, 37°C) and the amount of DNA released was measured by UV/Vis absorbance. In panels (A) and (C), data are presented as the average and standard deviation of multiple measurements made using three identically prepared films. In panel (B), data are presented as the average and standard deviation of multiple measurements made using two identically prepared films

Finally, we performed a series of experiments to characterize the behaviors of mixed multilayers having nominal structures that were the ‘inverse’ of those characterized above—that is, films having the structure of Film 4 in Figure 2, in which eight bilayers of polymer **2** and DNA were deposited onto a multilayer film composed of eight bilayers of polymer **1** and DNA [i.e., (polymer **1**/pEGFP)_8_(polymer **2**/pEGFP)_8_ films; the opposite of the procedure used to fabricate Film 3]. These films were fabricated on silicon substrates pre‐coated with a thin multilayer film (∼20 nm thick) composed of 10 bilayers of LPEI and SPS to facilitate the adsorption of polymer **1**/DNA layers, as described in past studies.^23^, ^27^ Figure 6A shows a plot of film thickness versus the number of polymer/DNA layers deposited, as determined by ellipsometry. These results reveal these ‘inverted’ mixed multilayers to grow in a linear manner and to final thicknesses (∼250 nm) similar to those exhibited by mixed Film 3 films (Figure 3A).

Additional characterization of these inverted mixed Film 4 multilayers revealed stark and substantial differences in DNA release profiles, compared to the behaviors of Film 3 multilayers, when these films were incubated in PBS at 37°C. Substrates coated with Film 4 multilayers also released DNA over a period of ∼60‐70 days (Figure 6B), but, in contrast to Film 3 multilayers (Figure 3B), exhibited nearly completely linear release profiles over that time period. Figure 6C shows the release profile of inverted mixed‐polymer/mixed‐plasmid multilayers having the structure of Film 7 in Figure 2 [i.e., (polymer **1**/pEGFP)_8_(polymer **2**/pRFP)_8_]. These results also reveal both plasmid constructs to be released at a constant rate over a period of ∼70‐80 days. These linear release profiles are very different from those exhibited by either (polymer **1**/DNA)‐type films^23^, ^27^ or (polymer **2**/DNA)‐type films^29^, ^31^ reported in past studies, or the mixed Film 3‐type and Film 6‐type multilayers reported above (Figure 3B,C). We performed cell transfection experiments similar to those described above and shown in Figure 5 using aliquots of erosion milieu collected during the erosion of the Film 7 coatings used to generate the results shown in Figure 6C. Unfortunately, the concentrations of DNA in samples collected during these long‐term, linear release experiments were sufficiently low to prevent meaningful conclusions regarding any potential differences in the rates of release of the pEGFP and pRFP plasmids from these inverted multilayer coatings.

## 4 Discussion

The iterative and step‐wise nature of the layer‐by‐layer assembly process used to fabricate the films investigated here provides a straightforward and practical framework for the design of thin films composed of multiple different layers of multiple different components (e.g., simply by varying the number of ‘layers’ of each component deposited during assembly).^18^, ^19^, ^42^, ^43^ This approach provides control over the relative loading of DNA that is incorporated into a film^17^, ^27^ and—provided that significant polymer interdiffusion and structural reorganization do not occur during or after assembly—can be used to design films with hierarchical nanoscale structures.^18^, ^19^ This general approach has thus been used in past studies to design DNA‐containing multilayers that promote either the simultaneous,^44^ staged/offset,^41^ or sequential release^30^, ^31^ or cellular expression^45^ of multiple different plasmid DNA constructs, with the types of DNA release profiles and other film behaviors that can be achieved generally depending heavily upon the structures of the cationic polymers that are used^5^, ^20^, ^24^, ^25^ and the presence (or absence) of intervening ‘barrier’ layers^30^, ^45^ that can prevent interdiffusion and mixing among layers.

Our group has reported extensively on the behaviors of multilayers fabricated using plasmid DNA and either poly(β‐amino ester)s (including polymer **1**)^5,^ 23, 27, 44 or charge‐shifting polymers (including polymer **2**)^5,^ 29, 31 as cationic building blocks, and on the influence of polymer structure and film architecture on the DNA release profiles of those materials.^5^, ^17^, ^20^, ^25^ The results of this current study demonstrate that this relatively limited pool of cationic materials can be used in combination to design conformal polymeric coatings that release DNA with new, useful, and unanticipated release profiles that cannot be attained using either type of polymer alone. The results shown in Figures 3B,C and 6B,C, for example, demonstrate that ‘mixed multilayer’ films fabricated using combinations of hydrolytically degradable and charge‐shifting polymer/DNA bilayers erode and release DNA in ways that differ substantially from those fabricated from polymer **1** or polymer **2** alone. Importantly, those results also reveal that the order in which the individual polymer layers in these films are deposited during assembly has a substantial influence on their subsequent DNA release profiles.

The deposition of hydrolytically degradable polymer **1**/DNA layers on top of charge‐shifting polymer **2**/DNA films (mixed Film 3 or Film 6 multilayers; Figure 2) leads to films with multi‐phase release profiles that are intermediate to those exhibited by polymer **1**/DNA and polymer **2**/DNA films, but that have elements that are otherwise common to both (e.g., an overall extended release profile similar to that of polymer **2**/DNA films, but an early‐stage release phase that is more reflective of the behavior of polymer **1**/DNA films). It is possible to interpret the profiles of these mixed Film 3 multilayers (Figure 3B,C) as being, to some extent, linear combinations of those exhibited by polymer **1**/DNA and polymer **2**/DNA films (e.g., a rapid, burst release of DNA characteristic of polymer **1**/DNA films, followed by an extended phase of release more typical of polymer **2**/DNA films). The results of experiments using otherwise identical films fabricated using pEGFP and pRFP plasmids (mixed Film 6 multilayers), however, suggest that these films do not simply consist of discrete ‘stacks’ of polymer **1**/DNA and polymer **2**/DNA bilayers (as represented schematically in Figure 2) that erode and behave independently of each other.

The EGFP and RFP expression results shown in Figure 5 and Figure S3, for example, suggest that pEGFP, which was deposited last during assembly, is released first (over a period of ∼48 hours), but that both pEGFP and pRFP are subsequently released simultaneously for prolonged periods after that initial burst phase. Those results, combined with the release profiles shown in Figure 3B,C, suggest that significant intermixing of individual polymer/DNA layers occurs in these materials, either (i) during layer‐by‐layer assembly or (ii) after they are exposed to physiologically relevant media. Interlayer diffusion and the ‘mixing’ of layers is well known to occur in many different polyelectrolyte multilayer systems,^18^, ^19^, ^46^, ^47^, ^48^, ^49^ and can be eliminated (or reduced/controlled)^47^, ^48^, ^49^ to design hierarchical films that can ‘stage’ the release of multiple agents by incorporating intermittent barrier layers during assembly,^30^, ^45^, ^47^ as noted above. It is likely that that same approach could be adopted here to design hierarchical films that promote the release of multiple plasmids with non‐overlapping release profiles. Although not investigated as part of this current study, our results suggest that the overall release profiles of these mixed Film 3‐type multilayers could also be tuned further, and over a broader range, by manipulating the relative numbers of polymer **1**/DNA and polymer **2**/DNA bilayers that are deposited during assembly (films constructed using eight bilayers of each type of polymer/DNA pair were used in all experiments in this study to explore feasibility and establish proof of concept).

Our results also suggest that the internal structures of these mixed multilayer assemblies—and thus their subsequent DNA release profiles—can be manipulated broadly and in useful ways simply by varying the order in which polymer **1**/DNA and polymer **2**/DNA bilayers are deposited during assembly. In contrast to the behaviors of mixed Film 3 multilayers, the deposition of charge‐shifting polymer **2**/DNA layers on top of hydrolytically degradable polymer **1**/DNA films (‘inverted’ mixed Film 4 or Film 7 multilayers; Figure 2) leads to films that exhibit linear release profiles for periods of up to ∼80 days (Figure 6B,C). These films do not exhibit multiple phases of release or other features that reflect linear combinations of the behaviors of fast‐releasing polymer **1**/DNA^23^, ^27^ and slow‐releasing polymer **2**/DNA^29^, ^31^ films. These results were unanticipated at the outset of these studies and, when combined, suggest (i) that interdiffusion also occurs in these ‘inverted’ (slow on top/fast on bottom) films, and (ii) that the extents and degrees of intermixing in these ‘inverted’ films are likely to differ substantially from those that occur in Film 3‐type (fast on top/slow on bottom) films.

Our current results suggest that the order in which polymer **1**/DNA and polymer **2**/DNA layers are deposited does not impact film growth profiles or thickness substantially (Figures 3A and 6A). We note also that the growth profiles exhibited by both of these types of films are linear in nature, and show no hints of ‘exponential‐type’ growth that is often associated with substantial diffusion of polyelectrolytes into and out of a multilayer during assembly (and that can lead to much thicker, but more compositionally homogeneous, films).^37^, ^38^, ^39^, ^40^ Additional physicochemical characterization, including characterization of potential changes in film morphology that could occur during these ‘change of polymer’ assembly processes, will be required to characterize the locations and dynamics of the polymers in these assemblies and understand the factors that contribute to the large differences in DNA release profiles reported here more completely. The results of this study do, however, provide guidance useful for the design of thin films that promote the extended, surface‐mediated release of DNA with linear release profiles that have been difficult to achieve using multilayers fabricated from other cationic polymer building blocks. The ability to fabricate these mixed multilayers on the surfaces of topologically complex objects, including clinical interventional devices such as intravascular stents (Figure 4), without substantial changes in DNA release profiles (Figure S2) suggests opportunities to further explore and exploit the potential utility of these new materials in many different applied biomedical and translational contexts.

## 5 Summary and conclusions

We have reported the fabrication and characterization of polymer‐based ‘mixed’ multilayer coatings fabricated using plasmid DNA and combinations of both hydrolytically degradable and charge‐shifting cationic polymer building blocks. Our results reveal (i) that films fabricated using combinations of polymers **1** and **2** exhibit DNA release profiles that are substantially different from films fabricated using DNA and either polymer **1** or polymer **2** alone, and (ii) that the order in which these two cationic polymers are deposited during assembly has a substantial impact on DNA release profiles when these materials are incubated in physiologically relevant media.

Mixed multilayers fabricated by depositing layers of polymer **1** and DNA onto multilayers composed of polymer **2** and DNA (Film 3‐type coatings) released functional plasmid DNA into solution over a period of ∼60 days, with multi‐phase release profiles that were intermediate to those of polymer **1**/DNA or polymer **2**/DNA films (i.e., a period of rapid release, followed by a second and more sustained period of release). In contrast, ‘inverted’ mixed multilayers fabricated by depositing layers of polymer **2** and DNA onto multilayers composed of polymer **1** and DNA (Film 4‐type coatings) yielded coatings with DNA release profiles that were almost completely linear over periods ranging from ∼60‐80 days. These results are consistent with substantial polymer layer interdiffusion in both types of mixed multilayer films, and suggest that the extent of intermixing in the ‘inverted’ mixed multilayers differs substantially from that present in Film 3‐type films. Additional support for the occurrence of layer interdiffusion in these materials was provided by the results of cell transfection experiments using DNA released from mixed multilayers fabricated using two different plasmid DNA constructs.

Thus, in addition to being ‘mixed’ by virtue of being composed of two different classes of cationic polymers that can promote film disruption by two completely different mechanisms, the multilayers reported here can also be regarded as being ‘mixed’ by virtue of substantial commingling and nanoscale mixing of their individual components that occurs either during assembly or after introduction to physiological media. Our results reveal this commingling of components to lead to new, useful, and unanticipated DNA release profiles, and expand the range of release profiles that can be accessed using a limited pool of layer‐by‐layer building blocks. Overall, the results of this study provide new approaches and guidance useful for the design of polymer‐based coatings that promote the surface‐mediated release of DNA with predictable release profiles, and suggest opportunities to design new classes of coatings for the localized and long‐term surface‐mediated delivery of DNA from topologically complex clinical interventional devices, including intravascular stents.

## Supporting information

Additional Supporting Information can be found in the online version of this article at the publisher's website.

**Figure S1.** Additional representative scanning electron microscopy images showing intravascular stents coated with Film 3 in regions where the multilayer film was delaminated from the substrate. These images were used, in addition to the image shown in Figure 4C of the main text, to estimate film thickness (∼240 nm ± 70 nm).
**Figure S2.** Plot showing the release of DNA from stainless steel stents coated with Films 1, 2, and 3. Films were incubated in PBS buffer (pH 7.4, 37°C) and the amount of DNA released into solution was measured by UV/Vis absorbance. Data are presented as the average and standard deviation of three identically prepared film‐coated stents.
**Figure S3‐A.** *Part of a three‐part figure; see companion images in Figures S3‐B and S3‐C for results arising from this extended‐release experiment at different time points*. Representative fluorescence microscopy images showing COS‐7 cells expressing GFP (green channel) and RFP (red channel) after treatment with samples of DNA collected during the erosion of substrates coated with Film 6 (additional results for samples collected during the erosion of substrates coated with Film 1 and Film 5 are also shown for comparison). The relative levels of GFP and RFP expression observed correspond qualitatively to the amount of each plasmid released from the film during the following time periods: 0‐1 hour, 1‐3 hours, 3‐6 hours, 6‐12 hours, 12‐24 hours, and 24‐48 hours. Scale bar = 250 μm.
**Figure S3‐B.** *Part of a three‐part figure; see companion images in Figures S3‐A and S3‐C for results arising from this extended‐release experiment at different time points*. Representative fluorescence microscopy images showing COS‐7 cells expressing GFP (green channel) and RFP (red channel) after treatment with samples of DNA collected during the erosion of substrates coated with Film 6 (additional results for samples collected during the erosion of substrates coated with Film 1 and Film 5 are also shown for comparison). The relative levels of GFP and RFP expression observed correspond qualitatively to the amount of each plasmid released from the film during the following time periods: 48‐72 hours, 3‐4.3 days, 4.3‐6 days, 10‐14 days, 14‐18 days, and 18‐22 days. Scale bar = 250 μm.
**Figure S3‐C.** *Part of a three‐part figure; see companion images in Figures S3‐A and S3‐B for* results *arising from this extended‐release experiment at different time points*. Representative fluorescence microscopy images showing COS‐7 cells expressing GFP (green channel) and RFP (red channel) after treatment with samples of DNA collected during the erosion of substrates coated with Film 6 (additional results for samples collected during the erosion of substrates coated with Film 1 and Film 5 are also shown for comparison). The relative levels of GFP and RFP expression observed correspond qualitatively to the amount of each plasmid released from the film during the following time periods: 26‐30 days, 30‐34 days, 34‐38 days, 38‐42 days, 42‐46 days, and 46‐50 days. Scale bar = 250 μm.
