# Supplementary material for: Controlling the surface‐mediated release of DNA using ‘mixed multilayers’
Source: Bioeng Transl Med. 2016 Aug 26;1(2):181–92. doi: 10.1002/btm2.10023 (PMC5125402; doi:10.1002/btm2.10023)
Supplement: Supplementary file 1 — Figure S1. Additional representative scanning electron microscopy images showing intravascular stents coated with Film 3 in regions where the multilayer film was delaminated from the substrate. These images were used, in addition to the image shown in Figure 4C of the main text, to estimate film thickness (∼240 nm ± 70 nm). Figure S2. Plot showing the release of DNA from stainless steel stents coated with Films 1, 2, and 3. Films were incubated in PBS buffer (pH 7.4, 37°C) and the amount of DNA released into solution was measured by UV/Vis absorbance. Data are presented as the average and standard deviation of three identically prepared film‐coated stents. Figure S3‐A. Part of a three‐part figure; see companion images in Figures S3‐B and S3‐C for results arising from this extended‐release experiment at different time points. Representative fluorescence microscopy images showing COS‐7 cells expressing GFP (green channel) and RFP (red channel) after treatment with samples of DNA collected during the erosion of substrates coated with Film 6 (additional results for samples collected during the erosion of substrates coated with Film 1 and Film 5 are also shown for comparison). The relative levels of GFP and RFP expression observed correspond qualitatively to the amount of each plasmid released from the film during the following time periods: 0‐1 hour, 1‐3 hours, 3‐6 hours, 6‐12 hours, 12‐24 hours, and 24‐48 hours. Scale bar = 250 μm. Figure S3‐B. Part of a three‐part figure; see companion images in Figures S3‐A and S3‐C for results arising from this extended‐release experiment at different time points. Representative fluorescence microscopy images showing COS‐7 cells expressing GFP (green channel) and RFP (red channel) after treatment with samples of DNA collected during the erosion of substrates coated with Film 6 (additional results for samples collected during the erosion of substrates coated with Film 1 and Film 5 are also shown for comparison). The relative levels o [file BTM2-1-181-s001.pdf]

Controlling the Surface-Mediated Release of DNA Using 'Mixed Multilayers'

Visham Appadoo,[1] Matthew C. D. Carter,[1] and David M. Lynn[1,2,*]

[1]Department of Chemistry, 1101 University Avenue, University of Wisconsin—Madison, Madison, Wisconsin 53706, and [2]Department of Chemical and Biological Engineering, 1415 Engineering Drive, University of Wisconsin—Madison, Madison, Wisconsin 53706. E-mail: dlynn@engr.wisc.edu

Supporting Information

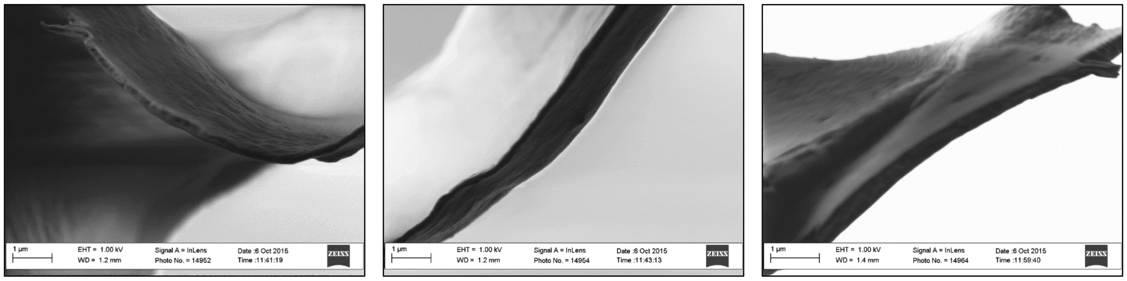

Figure S1. Additional representative scanning electron microscopy images showing intravascular stents coated with Film 3 in regions where the multilayer film was delaminated from the substrate. These images were used, in addition to the image shown in Figure 4C of the main text, to estimate film thickness (~240 nm ± 70 nm).

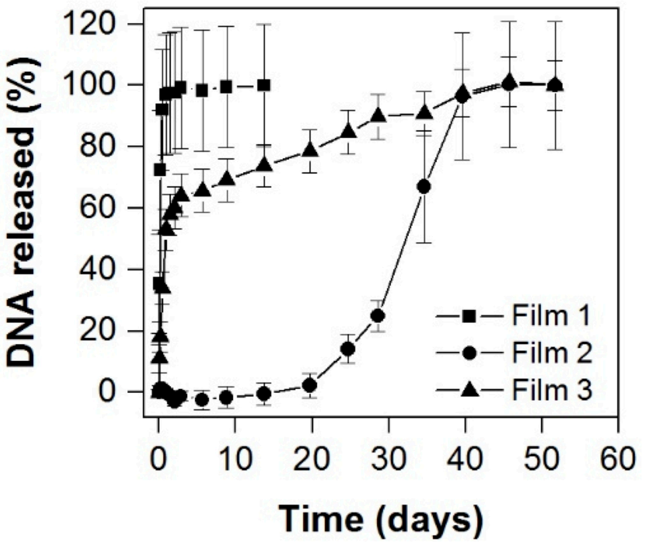

Figure S2. Plot showing the release of DNA from stainless steel stents coated with Films 1, 2, and 3. Films were incubated in PBS buffer (pH 7.4, 37 °C) and the amount of DNA released into solution was measured by UV/Vis absorbance. Data are presented as the average and standard deviation of three identically prepared film-coated stents.

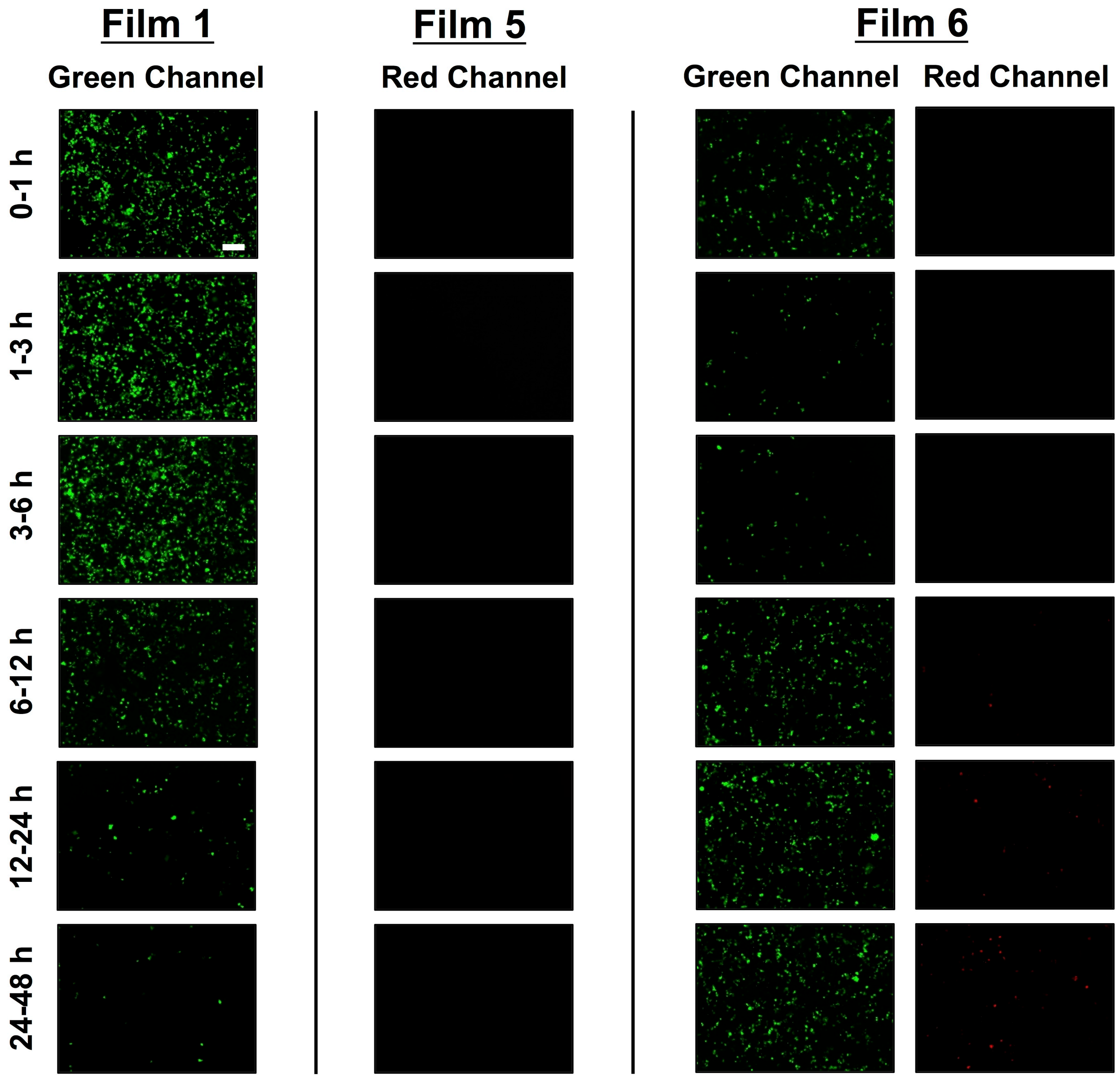

Figure S3-A. *Part of a three-part figure; see companion images in Figures S3-B and S3-C for results arising from this extended-release experiment at different time points.* Representative fluorescence microscopy images showing COS-7 cells expressing GFP (green channel) and RFP (red channel) after treatment with samples of DNA collected during the erosion of substrates coated with Film 6 (additional results for samples collected during the erosion of substrates coated with Film 1 and Film 5 are also shown for comparison). The relative levels of GFP and RFP expression observed correspond qualitatively to the amount of each plasmid released from the film during the following time periods: 0-1 hour, 1-3 hours, 3-6 hours, 6-12 hours, 12-24 hours, and 24-48 hours. Scale bar = 250 µm.

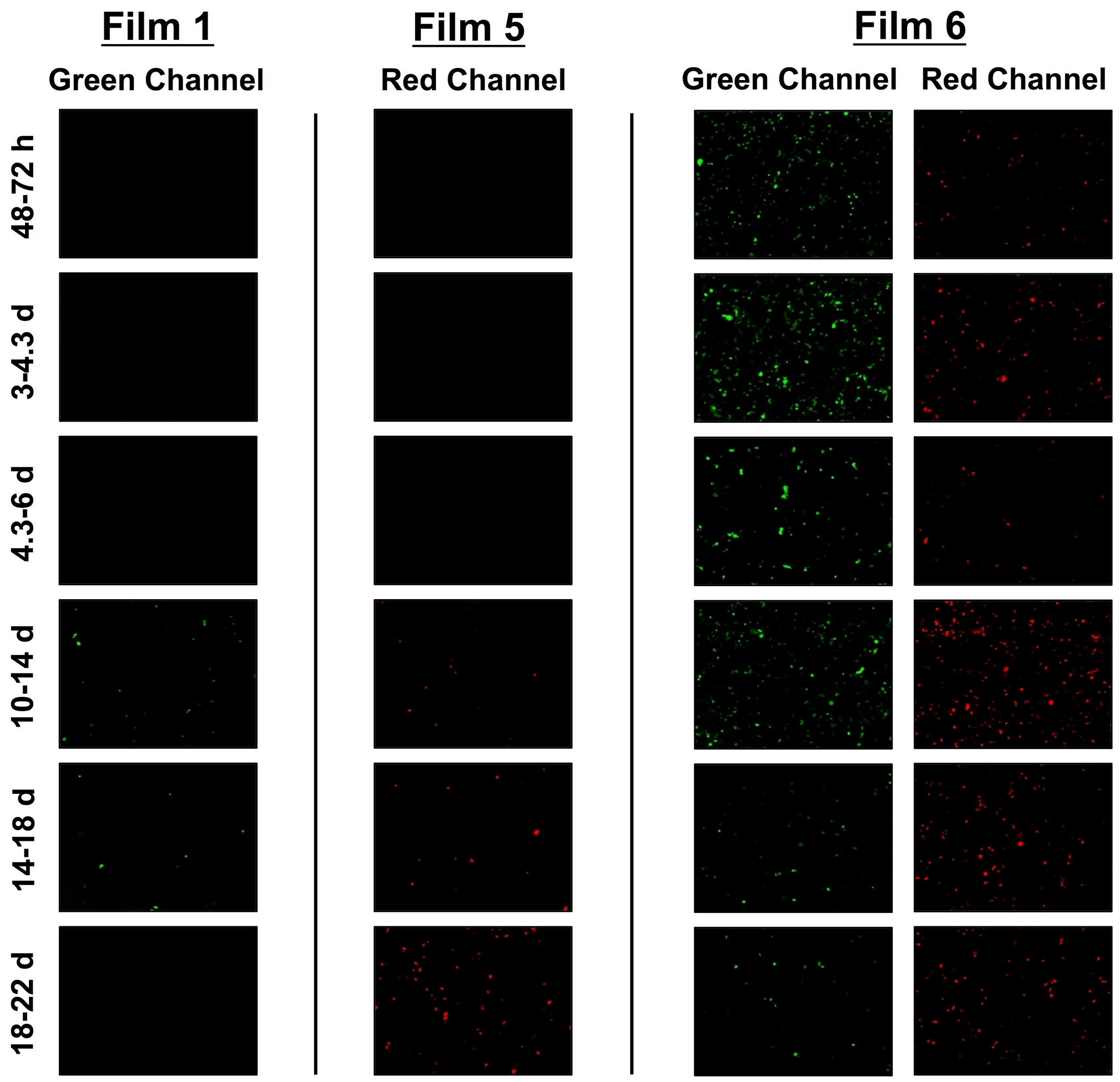

Figure S3-B. *Part of a three-part figure; see companion images in Figures S3-A and S3-C for results arising from this extended-release experiment at different time points.* Representative fluorescence microscopy images showing COS-7 cells expressing GFP (green channel) and RFP (red channel) after treatment with samples of DNA collected during the erosion of substrates coated with Film 6 (additional results for samples collected during the erosion of substrates coated with Film 1 and Film 5 are also shown for comparison). The relative levels of GFP and RFP expression observed correspond qualitatively to the amount of each plasmid released from the film during the following time periods: 48-72 hours, 3-4.3 days, 4.3-6 days, 10-14 days, 14-18 days, and 18-22 days. Scale bar = 250 µm.

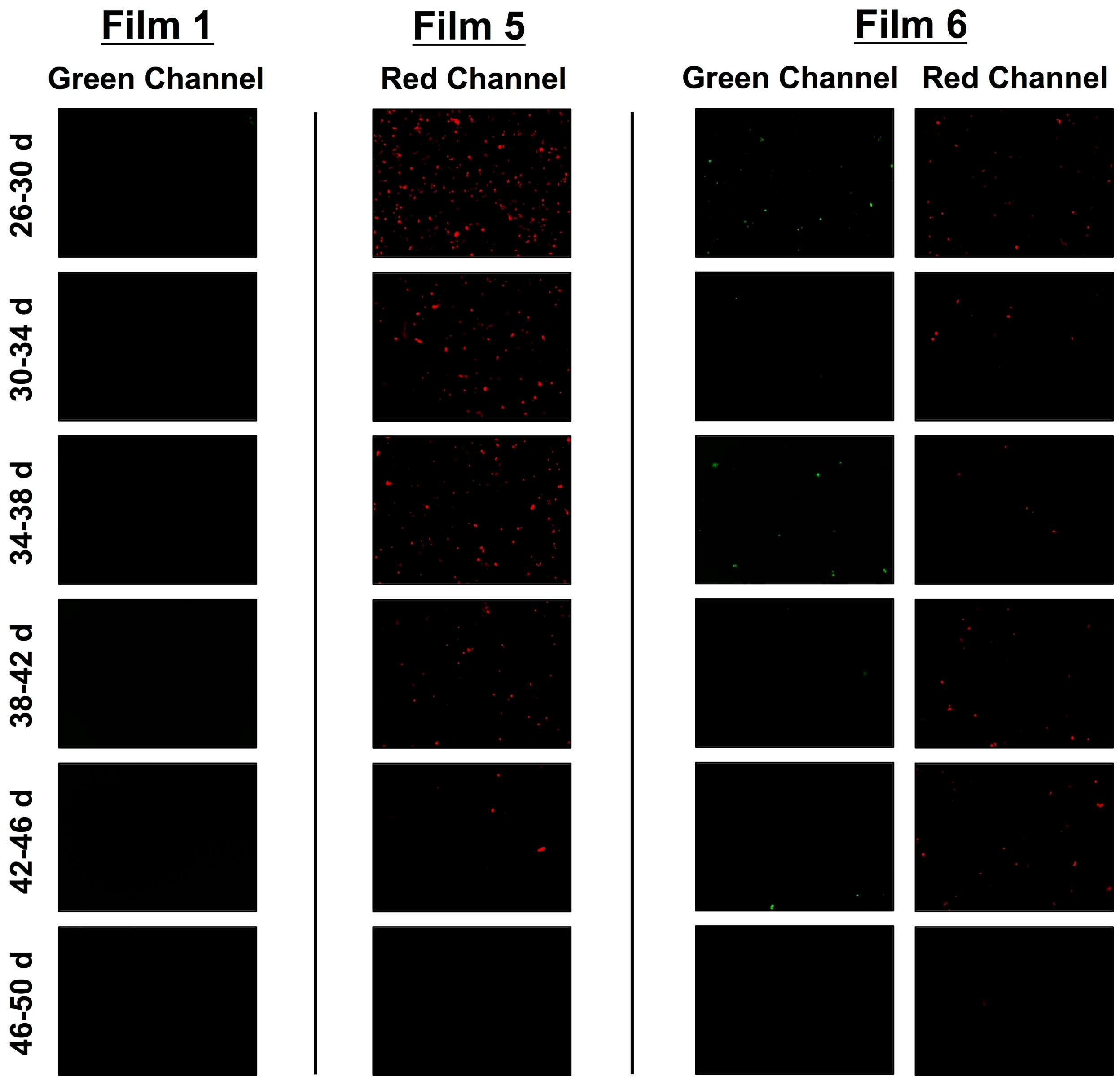

Figure S3-C. *Part of a three-part figure; see companion images in Figures S3-A and S3-B for results arising from this extended-release experiment at different time points.* Representative fluorescence microscopy images showing COS-7 cells expressing GFP (green channel) and RFP (red channel) after treatment with samples of DNA collected during the erosion of substrates coated with Film 6 (additional results for samples collected during the erosion of substrates coated with Film 1 and Film 5 are also shown for comparison). The relative levels of GFP and RFP expression observed correspond qualitatively to the amount of each plasmid released from the film during the following time periods: 26-30 days, 30-34 days, 34-38 days, 38-42 days, 42-46 days, and 46-50 days. Scale bar = 250 μm.
